# Stem Cell- and Cell-Based Therapies for Ischemic Stroke

**DOI:** 10.3390/bioengineering9110717

**Published:** 2022-11-20

**Authors:** Delia Carmen Nistor-Cseppentö, Maria Carolina Jurcău, Anamaria Jurcău, Felicia Liana Andronie-Cioară, Florin Marcu

**Affiliations:** 1 Department of Psycho-Neurosciences and Rehabilitation, Faculty of Medicine and Pharmacy, University of Oradea, 410087 Oradea, Romania; 2 Faculty of Medicine and Pharmacy, University of Oradea, 410087 Oradea, Romania

**Keywords:** ischemic stroke, neuroregeneration, neural stem cells, mesenchymal stem cells, extracellular vesicles, exosomes, miRNAs, clinical trials

## Abstract

Stroke is the second cause of disability worldwide as it is expected to increase its incidence and prevalence. Despite efforts to increase the number of patients eligible for recanalization therapies, a significant proportion of stroke survivors remain permanently disabled. This outcome boosted the search for efficient neurorestorative methods. Stem cells act through multiple pathways: cell replacement, the secretion of growth factors, promoting endogenous reparative pathways, angiogenesis, and the modulation of neuroinflammation. Although neural stem cells are difficult to obtain, pose a series of ethical issues, and require intracerebral delivery, mesenchymal stem cells are less immunogenic, are easy to obtain, and can be transplanted via intravenous, intra-arterial, or intranasal routes. Extracellular vesicles and exosomes have similar actions and are easier to obtain, also allowing for engineering to deliver specific molecules or RNAs and to promote the desired effects. Appropriate timing, dosing, and delivery protocols must be established, and the possibility of tumorigenesis must be settled. Nonetheless, stem cell- and cell-based therapies for stroke have already entered clinical trials. Although safe, the evidence for efficacy is less impressive so far. Hopefully, the STEP guidelines and the SPAN program will improve the success rate. As such, stem cell- and cell-based therapy for ischemic stroke holds great promise.

## 1. Introduction

Due to its high incidence, ranging between 100 and almost 300/100,000 persons/year, stroke poses a heavy burden on healthcare systems worldwide. Epidemiological studies have revealed that despite the slight decline in incidence rates in 2016 compared to 1990 achieved through the vigorous implementation of prophylactic measures, the actual number of people experiencing a first ever stroke annually has almost doubled, and that about 60% of patients are younger than 70 years [1]. The total number of disability-adjusted life-years lost because of stroke has also increased by 20% in the same time-period, leading to stroke being escalated as the second cause of disability worldwide [1]. Considering the trend towards the aging of the global population, and the linear relationship between stroke incidence and age [2], as well as the increasing prevalence of well-established vascular risk factors such as obesity [3] and diabetes mellitus [4], the number of patients living with stroke aftermath will likely increase.

Although recanalization therapy has been a significant breakthrough in acute ischemic stroke care, only a small percentage of patients meet the eligibility criteria [5]. For a long time, the major barrier in applying revascularization therapies was the long or unknown time elapsed since stroke onset. However, more recent studies using perfusion imaging (EXTEND trial) have suggested the possibility of extending the time window to 9 h for intravenous thrombolysis with recombinant tissue plasminogen activator, and mechanical thrombectomy may be performed up to 24 h after stroke onset, a recommendation based on the results of the DAWN trial [6]. Additional criteria, such as stroke severity and the ASPECTS (Alberta Stroke Program Early CT Score) score should be taken into consideration [6]. Nonetheless, in the various trials reporting the results of these methods, up to 60% of patients undergoing a recanalization procedure do not achieve functional independence [7]. Since neurons are post-mitotic non-dividing cells, and given the limited ability of the central nervous system (CNS) to self-repair, alternative methods to promote tissue regeneration, such as stem cell- or cell-derived therapies, may successfully complement traditional rehabilitation methods based on promoting neuroplasticity or enhancing the production of growth factors, and they could revolutionize stroke treatment.

In the following sections we will discuss the mechanisms of cell destruction after ischemic stroke and the signaling cascades involved, and we highlight the potential applications of stem cell- and cell-based therapies after ischemic stroke.

### Search Strategy and Selection Criteria

The references cited in this review have been obtained from the PubMed and Google Scholar databases. We referenced full-text review articles, experimental studies, randomized control trials, and meta-analyses. No limits were used.

## 2. Cell Loss after Acute Ischemic Stroke

For decades, it has been known that the cerebral infarcted area can be divided into an infarcted core, where cells become rapidly necrotic, and a surrounding penumbral area, where cells are subject to lower levels of ischemia and maintain viability for a limited amount of time, although they are functionally depressed [8]. Traditionally, it has been considered that in the severely oxygen- and glucose-deprived core, cell death occurs through necrosis affecting neurons, glial cells, and endothelial cells; while in the penumbra, the process is more delayed and occurs mainly through apoptosis [5]. However, recent research has revealed many types of cell death, with characteristic morphological changes and mediations through specific pathways, which could be selectively inhibited [8].

It is generally accepted that in the infarct core, the massive deprivation of oxygen and glucose causes a depletion of ATP, followed by the failure of the sodium pump. The cells are persistently depolarized, with the opening of the voltage-gated sodium and calcium channels, and the activation of the N-methyl-D-aspartate receptors (NMDARs), which further augment intracellular Na^+^ and Ca^2+^ concentrations, leading to increased osmotic pressure. The high intracellular Ca^2+^ levels also activate a series of enzymes (phospholipases, calpains, and proteases) which break down the cytoskeleton. The cells swell, and ultimately their plasma membrane ruptures, allowing for the cellular contents (including neuromediators) to be released into the interstitial space, and for glutamate to bind to NMDARs on neighboring cells and at distant sites, propagating these vicious cascades. This type of cell death is referred to as “oncosis”, and affects neurons, as well as astrocytes or oligodendrocytes [9].

**Figure 1:**
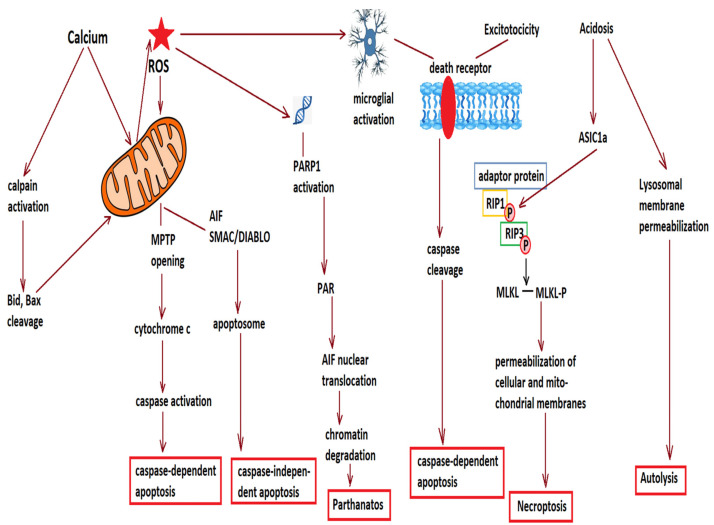
Various mechanisms contribute to cell loss in the penumbral area. Reactive oxygen species (ROS) and increased cytosolic calcium damage mitochondria, and lead to opening of the mitochondrial permeability transition pore (MPTP) and release of cytochrome c and apoptosis inducing factor (AIF). Increased calcium also activates calpains, which cleave pro-apoptotic factors such as Bid and Bax, promoting their mitochondrial translocation and further permeabilization of the mitochondrial membrane. Cytochrome c activates the caspase cascade and leads to caspase-dependent apoptosis, while AIF and second mitochondrion-derived activator of caspase/direct inhibitor of apoptosis-binding protein with low pI (SMAC/DIABLO) activate caspase-independent apoptosis. ROS also damage DNA and activate poly(ADP-ribose) polymerases (PARP), leading to the production of poly(ADP-ribose) or PAR, which promotes the nuclear translocation of AIF and chromatin degradation, leading to parthanatos. Another consequence of ROS production is microglial activation, which together with excessive stimulation of N-methyl-D-aspartate receptors (excitotoxicity) activate the membrane death receptors, leading to caspase cleavage, as well as to receptor interacting kinase 1 (RIP1) phosphorylation, followed by the phosphorylation of RIP3 and mixed lineage kinase domain-like (MLKL), leading to oligomerization of phosphorylated MLKL at plasma membranes and cell rupture (necroptosis). Acidification, common in the penumbral area of cerebral infarction, promotes the association of acid-sensing ion channel 1a (ASIC1a) with RIP1 and the activation of the latter. In addition, an acidic environment, together with calpain activation, permeabilizes the lysosomal membrane and allows for the release of lysosomal proteases, cathepsins, and hydrolases into the cytosol, leading to autolysis.

In the surrounding penumbral area, glucose and oxygen deprivation is less impressive, allowing for a limited amount of ATP to be generated, but which will be used in a series of pathways executing cell death in a delayed and programmed manner. Glutamate binding to NMDARs and other glutamate receptors increases the intracellular Ca^2+^ concentrations, which overload the mitochondria, leading to the opening of the mitochondrial permeability transition pore (MPTP) and the cytoplasmic release of cytochrome c and other apoptotic factors, which initiate caspase-dependent and -independent apoptosis processes [10]. In addition, the activation of BH3-only proteins (a group of the pro-apoptotic Bcl-2 family proteins) causes Bax activation and translocation to the mitochondria, where it oligomerizes and forms pores on the outer mitochondrial membrane, allowing further cytochrome c release in the intrinsic or mitochondrial apoptosis pathway [7]. Cellular destruction releases damage-associated molecular patterns (DAMPs), which activate the microglia [11], leading to the binding of tumor necrosis factor (TNF)-α to the cell surface, the recruitment of Fas-associated death domain (FADD), and the subsequent activation of pro-caspase 8, which initiates the autolytic process of extrinsic apoptosis [12]. However, other pathways leading to cell death have been described and characterized. For example, necroptosis is a form of regulated necrosis for which RIP1 (receptor interacting kinase 1) phosphorylates and activates RIP3, leading to the expression of mixed lineage kinase domain-like (MLKL) that will phosphorylate and oligomerize at the plasma membrane, and lead to the rupture of the cell membrane [13]. Acidosis, as that which occurs after stroke, induces the association of RIP1 with acid sensing ion channel 1a (ASIC1a) and activates RIP1 [14]. Apoptosis inducing factor (AIF), released from mitochondria following the opening of the MPTP, translocates to the nucleus and degrades chromatin, activating poly(ADP-ribose) polymerases (PARP). The product of PARP, poly(ADP-ribose), or PAR inhibits hexokinase and causes bioenergetic failure, leading to a specific form of cell death called “parthanatos” [7,15]. Ischemia-induced increases in cytosolic Ca^2+^ also activate calpain 1, which increases the permeability of the lysosomal membrane and leads to lysosomal cell death or “autolysis” [16]. Finally, the loss of neurons in the infarcted area will lead to the death of other, distant neurons that are connected to the former ones through a process known as “transneuronal degeneration” [7], because normal synaptic activity, through the activation of NMDARs, suppresses the activity of apoptotic factors such as Puma, APAF-1, or caspase-9 [17]. Figure 1 summarizes the various types of cell death through which cells are lost in the penumbral area surrounding the infarcted core.

## 3. Stem Cell Therapies for Ischemic Stroke

Starting with the pioneering research of Sharp and colleagues, who demonstrated the survival of fetal neocortical grafts in adult rat cortex subject to ischemia [18], research on stem cell therapy in stroke has made considerable advances. Initially used as replacement therapy for lost cells, it is currently accepted that stem cells can secrete therapeutic substances that interfere with many pathogenic cascades and promote survival, migration, differentiation, and the functional integration of grafted cells into the brain circuitry [19].

### 3.1. Types of Stem Cells

A variety of stem cells have been tested in animal models of ischemic stroke, as well as in neurodegenerative disease models [20,21].

#### 3.1.1. Neural Stem Cells

Neural stem cells (NSCs) can be derived either from the inner cell mass of the blastocyst between days 5 and 7 post-conception (before implantation into the uterus), in which case they are omnipotent embryonic stem cells (ESCs) that can differentiate into NSCs, or from the fetal nervous system, are harvested between 7 and 21 days after conception, in which case they are multipotent neural stem cells and can differentiate into neurons, astrocytes, and oligodendrocytes. Fetal-derived NSCs are obtained by dissociating the human fetal cortex, spinal cord, or mesencephalon [22] and culturing them in a mitogen-rich environment. In the presence of epidermal growth factor (EGF) and basic fibroblast growth factor (bFGF), the cells tend to develop a neurospheric culture, the cellular assemblages needing to be mechanically or enzymatically dissociated and replated, repeating the procedure several times in order to prevent the formation of large clusters of cells that are at risk of undergoing necrosis due to a lack of nutrients in the center of the neurosphere [23]. Alternatively, NCSs can be cultured in serum-free medium supplemented with EGF and/or bFGF, in the presence of a substrate such as laminin, poly-L-ornithine, or fibronectin, leading to an adherent monolayer culture of cells [24].

NSCs have several advantages over other stem cell types due to: (1) high brain-like similarity [25]; (2) a low rejection rate, even for inter-individual or inter-species transplanted cells NSCs compared to neuronal cells [26,27], and (3) significant chemotaxis, with NSCs being able to migrate to the site of damage [28]. Research has shown that chemokine stromal cell-derived factor-1 alpha (SDF-1α/CXCL12), increased in areas of ischemia, interacts with NSC CXCR4 receptors and mediates their migration to sites of injury [29]. As such, they can replace lost neural cell types, but are also able to produce neuroprotective and regenerative growth factors [30]. NSCs maintain their multipotent differentiation potential but tend to undergo early senescence [31], a setback that can be overcome through genetic modification and immortalization of the cell line. The most promising approach appears to be fusion of the conditional immortalized *C-MYC* gene (avian myelocytomatosis viral oncogene homolog) with a mutated estrogen receptor, leading to the CTX0E03 human NSC line [32], which showed efficacy in promoting behavioral recovery in stroke patients [33]. Another cell line, NSI-566, derived from human fetal spinal cord and authorized by the Food and Drug Administration of the USA for clinical trials, has been used in nine chronic stroke patients with encouraging results regarding the recovery of motor function, but further characterization of the grafted tissue and confirmation of the results via double-blind controlled trials is required [34]. Adult neural stem cells could, at least in theory, be harvested from the subventricular and the subgranular zones of the human brain. However, their numbers decrease with age, and culturing them in vitro alters the cell state and favors immune rejection [26].

Takahashi and Yamanaka managed to successfully reprogram adult somatic cells and obtain gene-matched induced pluripotent stem cells (iPSCs) through the retroviral transduction of the sex-determining region Y-box and the octamer-binding transcription factor 4 (2 transcription factors), and C-MYC and the Kruppellike factor 4 (two signaling molecules) [35]. Further, in the presence of specific proteins and inducers, iPSCs can differentiate into NSCs [36]. Unfortunately, the obtained iPSCs may have chromosomal aberrations, and they have tumorigenic and immunogenic potential [21,37]. The transdifferentiation of differentiated and proliferating non-neuronal cells (such as astrocytes or pericytes) into neuronal cell lineages is the cheapest method of obtaining NSCs, and it can be achieved through the retroviral-mediated co-expression of several transcription factors [38]. This approach bypasses the pluripotent cellular stage and diminishes the risk of neoplasia [39]. However, the use of NSCs raises many ethical, religious, and even scientific issues. Moreover, the moratorium on the uses of federal funds for embryonic stem cell research in the 1990s in the USA seriously hindered scientific advances. The first clinical trial using human neuroteratocarcinoma cells transformed into neurons for post-stroke treatment was abandoned due to the financial constraints of the sponsor company [19,40].

#### 3.1.2. Mesenchymal Stem Cells

Mesenchymal stem cells (MSCs) can be derived from adipose tissue, tooth buds (from adult or embryonic sources), bone marrow, liver; or umbilical cord, cord blood, and placenta [21]. Bone marrow-derived cells have been well-characterized and comprise MSCs, mononuclear cells (MNCs), multipotent adult progenitor cells (MAPCs), endothelial progenitor cells, SB623, and multilineage-differentiating stress-enduring cells (Muse) [41,42,43,44]. Human placenta-derived and human amnion epithelial cells appear to diminish the magnitude of the inflammatory response in the early phase [20], while human bone marrow endothelial progenitor cells are able to repair the BBB in rats [45].

In the acute phase of rat stroke models, MSCs inhibited neuronal apoptosis, protected mitochondrial function, and reduced microglial activation [46], while in the subacute phase, human MSCs promoted angiogenesis, reduced blood-brain barrier disruption, and inhibited the release of pro-inflammatory cytokines and M2 to M1 microglial phenotype shift [47]. Angiogenesis and neurogenesis were augmented by human umbilical cord-derived MSCs, even if they were transplanted in the chronic phase of stroke in rat models [48]. However, few of the transplanted MSCs differentiated into mature cells [49]. Much of the beneficial effects of MSCs appear to be mediated by the wide range of neuroprotective factors that they are able to produce when exposed to extracts from the ischemic brain, such as nerve growth factor (NGF), brain derived neurotrophic factor (BDNF) or vascular endothelial growth factor (VEGF), or chemokines such as CXCL12 [20,50]. The genetic engineering of these cells can further enhance their ability to produce these beneficial molecules, increasing their efficacy [51,52]. The beneficial effects of bone marrow-derived stromal cells can also be augmented through exposure to a hypoxic environment (hypoxic preconditioning), as shown by Chen and colleagues [53].

#### 3.1.3. Cell-Derived Vesicles

To bypass a series of side effects of cell delivery, extracellular vesicles (exosomes and microvesicles) can be used with similar effects. They have a bilayer membrane and contain proteins, microRNAs (miRNAs), messenger RNAs (mRNAs), and DNA and lipids, being able to influence the immune response, cell differentiation, and tissue repair and angiogenesis [54]. For the formation of the exosome, the plasma membrane initially bulges inward and forms an intracellular vesicle (the endosome), which subsequently fuses with the plasma membrane and empties its contents into the extracellular space [55]. Microvesicles bud directly from the plasma membrane, are larger than exosomes, contain cytosolic proteins, lipids, and mRNAs and miRNAs as well, and they are internalized by the recipient cell following ligand–receptor interaction [36,56]. These cell-derived vesicles have the advantages of low immunogenicity, a low risk of vessel thrombosis following transplantation, and they are able to cross the BBB. In addition, the miRNAs contained can be easily genetically modified and large-scale production of extracellular vesicles is possible at a reasonable cost [57].

### 3.2. Mechanisms Involved in the Therapeutic Effects of Stem Cells

Differentiation into nerve cells, the reconstruction of synapses, and integration of the transplanted cells into neural networks cannot be accomplished in a short period of time to explain the efficacy of NSC transplantation in the acute phase of stroke [58]. In fact, stem cells have a multimodal action, protecting endangered neural cells in the acute phase, promoting proliferation of endogenous neural stem cells in the subventricular and subgranular zone, fostering synaptic pruning and remodeling, and promoting angiogenesis [30] in later stages. These effects are believed to be mediated by extracellular vesicles [59]. A series of miRNAs such as miRNA-9 regulating axonal regeneration, miRNA-200b mediating myelin expression [60], miRNA-17-92, which activates signaling pathways for neuronal growth [61], or miRNA-15a, activating angiogenesis in brain tissues, have all been detected in exosomes from the cerebrospinal fluid [26].

#### 3.2.1. Modulation of the Immune Response

Cellular injury in ischemic stroke causes the release of damage-associated molecular patterns (DAMPs) that activate microglia, leading to the secretion of pro-inflammatory cytokines such as interleukin (IL)-1β, tumor necrosis factor-alpha (TNF-α), or Il-6 within minutes after the onset of acute oxygen and glucose deprivation [11], as well as astrocytes, which proliferate rapidly and secrete pro-inflammatory cytokines, chemokines, and metalloproteinases [57]. The released cytokines upregulate the expression of chemokines such as chemokine ligand 1 (CXCL1) or monocyte chemoattractant protein-1 (MCP-1 or CC β ligand 2) on endothelial cells, leading to infiltration of the injured tissue with peripheral monocytes/macrophages, and further exacerbation of the inflammatory response [62]. Although in the subacute and chronic phases of stroke, the neuroinflammatory response promotes tissue regeneration, blocking the acute phase reduces the magnitude of tissue injury caused by the occlusion of a cerebral artery [11].

Through the release of nerve growth factor, brain derived neurotrophic factor or glial-derived neurotrophic factor (GDNF), transplanted NSCs inhibit this pro-inflammatory cascade and, thus, curtail the secondary injury cascade [63], a mechanism also known as the “bystander effect”. The intraparenchymal transplantation of fetal NSCs and iPSC-NSCs in a rodent stroke model was followed by the decreased expression of IL-1β, IL-6, TNF-α, intracellular adhesion molecule 1 (ICAM1), vascular adhesion molecule 1 (VCAM1), and MCP-1 [64]. Fetal NSCs more effectively reduced infarct volume compared to iPSC-NSCs, probably due to the differences in the capacity of neurotrophic signaling [30]. The “bystander effect” can be obtained via the intravenous delivery of NSCs as well, although targeting them to the brain parenchyma increases their immunomodulatory capacity [65]. Similar effects can be achieved through the delivery of exosomes containing miRNA-126 [66], while miRNA-124-3p in EVs derived from microglial cells shifts the microglial phenotype from the pro-inflammatory M1 one to the anti-inflammatory M2 phenotype [67], promoting tissue regeneration during later stages.

The effect of stem cells from a single source is, however, limited, and the transplantation of mixed components or stem cells associated with stem cell additives is advocated for enhancing their anti-inflammatory effects in the acute phase of stroke [68,69].

#### 3.2.2. Cell Replacement and the Homing of Transplanted Stem Cells

After transplantation through various routes (intravenous, intra-arterial, or transnasal delivery), cells move to the site of the injury by utilizing chemotaxis [26], a process known as “homing”. A series of signaling factors, such as stromal derived factor-1/CXC chemokine receptor type 4, or monocyte chemotactic protein-3/cinnamoyl-coenzyme A reductase promote stem cell migration, while the subsequent activation of integrin β1 leads to the adhesion of transplanted cells [70]. Once at the site of the damaged tissue, transplanted stem cells undergo a series of processes that may extend up to 6 months after the acute stroke [26]. Endogenous NSC proliferation in the subventricular zone and dentate gyrus, as well as the migration of these cells to the region of tissue damage, was also enhanced after NSC transplantation in the first 2 days after stroke onset [71]. The effect is, again, likely mediated by neurotrophic factors that are secreted by the transplanted cells [30,72].

#### 3.2.3. Establishment of Neuron Polarity and Cell Division

Once at the injury site, NSCs recapitulate the processes occurring during neurogenesis within 7 days, undergoing division and establishing neuron polarity. Division may be symmetrical, leading to two neural progenitor cells, or asymmetrical, leading to a neuron and a neural progenitor cell [73]. Polarity is the result of the spatial arrangement of cells during division, and is regulated by protein complexes, as well as by regulatory genes [74]. Radial glial cells are polarized along the apical–basal axis, and serve as scaffolds for migrating neurons.

#### 3.2.4. Vascular Regeneration

Reestablishing the neurovascular unit (neurons, pericapillary microglia, astrocytes, basal lamina, pericytes, and endothelial cells) as early as possible is of crucial importance for rescuing endangered cells in the infracted area. Research has shown that neurovascular regeneration occurs within 4 to 7 days after the ischemic insult [26], and is largely dependent on VEGF and bFGF signaling [75]. Moreover, transplantation of superoxide dismutase-overexpressing NSCs enhanced angiogenesis [76], as did the transplantation of cells of the CTX0E03 line [77]. Hypoxic preconditioned NSCs upregulate the expression of miRNA-210, which has been shown to enhance neurogenesis and angiogenesis in mice [78,79].

#### 3.2.5. Neuroregeneration and Neurite Growth

Synaptic pruning and synaptic rewiring are the two main steps of synaptic regeneration [80], which occur within 1 month after stroke [26]. A series of substances secreted by astrocytes and differentiated NSCs promote neurogenesis, while neurite outgrowth is dependent on VEGF, thrombospondin 1 and 2, and bFGF and EGF released by the transplanted stem cells [81,82]. Further, BDNF released by NSCs differentiated into glial cells promote dendrite growth and myelination [83], while axonal growth is dependent on the axonal growth cone protein GAP-34 expressed by the transplanted cells [30].

#### 3.2.6. Myelination

Myelin repair, necessary for re-establishing the normal function of the neural network, is a process extending over 3 months after stroke, and it consists of myelination of the bare axons, removal of the damaged myelin sheaths, replacement by new internode segments, and remodeling of existing sheaths [84]. It occurs through the recruitment and differentiation of oligodendrocyte precursor cells, although mature neurons can also promote myelin repair, at least in zebrafish models [85].

#### 3.2.7. Synaptic Rewiring and Remodeling of Brain Circuits

Restoring brain function after an injury needs the brain network to be reconstructed, a process that continues up to 6 months after stroke and requires the differentiation of neurons, the growth of neurites, and myelin repair [58]. Dendritic spines bind to axonal veins and form “potential synapses” in the cortical columns, while new synapses are formed between pre- and postsynaptic neurons and dendritic spines [86], the strength of which changes dynamically over time. Neurons derived from transplanted stem cells are incorporated into neural circuits [87]. Several signaling molecules regulate the remodeling of the neural network: while the myelin-associated protein, the paired immunoglobulin-like receptor B and Nogo receptor are enhancing synaptic plasticity [26], myelin-associated glycoprotein and oligodendrocyte myelin glycoprotein inhibit synaptic remodeling [88].

**Figure 2:**
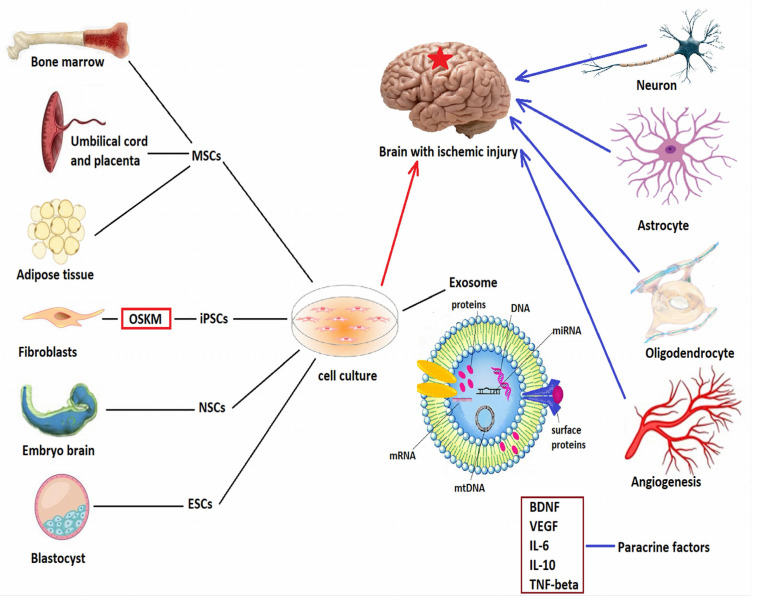
Embryonic pluripotent stem cells (ESCs) are derived from the inner layer of the blastocyst, Neural multipotent stem cells are obtained from human fetal cortex, mesencephalon, or spinal cord. Mesenchymal multipotent stem cells can be harvested from bone marrow, umbilical cord and placenta, or from adipose tissue. There is also the possibility of obtaining induced pluripotent stem cells (iPSCs) via transduction of the four OSKM genes: octamer-binding transcription factor 4 (Oct4), sex-determining region Y-box 2, (Sox2), the Krupellike factor 4 (Klf4), and the avian myelocytomatosis viral oncogene homolog (c-myc). Once harvested, stem cells are cultured in special culture medium, where they release exosomes carrying proteins, DNA, messenger RNA (mRNA), microRNAs (miRNA), and mitochondrial DNA (mtDNA). Once delivered to the brain by various routes, they differentiate into neurons, astrocytes, and oligodendrocytes, and release various growth factors (such as brain derived neurotrophic factor-BDNF or vascular endothelial growth factor-VEGF) and anti-inflammatory cytokines (interleukins IL-6, IL-10, or tumor necrosis factor β), which modulate neuroinflammation and promote angiogenesis, neurogenesis, neural differentiation, and synaptogenesis.

Figure 2 provides a schematic overview of the sources and mechanisms of action of stem cells in neuroregeneration.

### 3.3. Stem Cells in the Experimental Therapy of Cerebral Ischemia

#### 3.3.1. In Vitro Experimental Studies

In vitro studies have paved the way for translating the findings into animal models of ischemic stroke and even to clinical trials, as will be discussed further. Using the oxygen and glucose deprivation model, Huang and colleagues showed that neural N17 cells cocultured with MSCs display reduced apoptosis and decreased TNF-α levels [89], restoring their proliferation rates. Similar results have been obtained by co-culturing oxygen- and glucose-deprived cells with supernatants from the MSCs culture that are rich in extracellular vesicles [90].

Furthermore, elevated levels of growth factors lead to the activation of signaling mechanism for neuronal survival, such as the mitogen activated protein kinase (MAPK)/ extracellular signal-regulated kinase ½ (ERK1/2) cascade [91], or the phosphoinositide 3-kinase (PI3K)/serine/threonine kinase 1 (Akt) pathway [92]. Another pathway that is influenced by the paracrine factors released by stem cells is the c-Jun-terminal kinase (JNK) pathway, which is important in neuronal apoptosis following ischemic stroke, and which is inhibited by these paracrine factors [93]. In addition, after MSC delivery, the Wnt/β-catenin pathway, an evolutionary conserved pathway that orchestrates BBB maturation during ontogenic development, is activated and prevents BBB breakdown [94].

#### 3.3.2. Studies on Stem Cell Therapy after Ischemic Stroke in Animal Models

Research focusing on the neurorestorative effects of stem cell transplantation after permanent or transient ischemic stroke in animal models is rapidly expanding. Both NSCs and MSCs from various sources have been used in many settings. Huang and colleagues found 199 animal studies using NSCs for ischemic stroke published between 2004 and 2018, but only 62 gave complete information on the study protocol, inclusion criteria, and measures of outcome, and were included in a meta-analysis study [95]; while Zheng and colleagues reported on 78 studies selected from 421 publications on MSCs published between 2008 and 2017 [96]. NSCs can migrate into the peri-infarct area and differentiate into neural cell types, while MSCs act rather through the “bystander effect’.

For NSC transplantation, it appeared that transplantation within the first 7 days after the ischemic insult (in the acute or subacute stage) led to greater reduction in infarct size, likely through the inhibition of apoptosis and secondary tissue damage, and the preservation of neural circuits [95,97]. The meta-analysis also revealed that relatively low doses of below 1 × 10^6^ cells/kg delivered into the brain parenchyma resulted in improved functional recovery, probably due to free migration to the lesion site and the avoidance of exacerbation of brain ischemia [95]. In addition, allogeneic transplantation was more efficient for lesion size reduction compared to xenotransplantation, a finding that has been explained more likely by immunological reactions [98]. Interestingly, autologous stem cells translated into better structural outcomes, while allogeneic cell transplantation led to better functional improvement [99]. The time from the onset of ischemia and stem cell transplantation varied between 10 min [100] and 3 weeks [101]. Tumorigenesis is a possible side effect of NSC transplantation; 30 studies included in the meta-analysis by Huang et al. reported on this negative outcome and claimed no malignancy formation, confirming that stem cells are a safe alternative for stroke treatment, as previously suggested [102]. A brief and simple overview of the studies serving for the meta-analysis of NSCs in animal models of stroke analyzed by Huang and colleagues is shown in Table 1.

Mesenchymal stem cells lack HLA-II molecules, meaning that they are less immunogenic, and are pluripotent cells obtained from adult tissues, which does not raise ethical issues, making these cell lines the preferred ones for the pre-clinical and clinical settings [103]. Aside from the intraparenchimatous route, they can be administered less invasively, via intravenous, intra-arterial, intranasal, or intrathecal delivery. However, following intravenous delivery, most of the transplanted cells tend to accumulate in the liver, spleen, kidney, and especially in the lung [104]; only 4% of the delivered number of cells are located in the ischemic brain tissue [103]. The intra-arterial route leads to higher degrees of functional recovery, but carries the risk of cerebral microvascular embolism, the formation of intra-arterial emboli, and may further decrease local cerebral blood flow—side effects related to the dose of injected cells [105]—while the intranasal delivery route can lead to similar effects as intracranial administration [106], and can be repeated. However, the human olfactory bulb is smaller than in rodents used for stroke models.

In the meta-analysis of Zheng and colleagues [96], MSCs were administered within 24 h from the acute ischemia in 53 studies, which is difficult to translate into human studies. Another meta-analysis published by Lalu and colleagues [107] excluded the studies in which stem cells were administered before 3 days after stroke onset, and analyzed 76 out of 302 preclinical studies published between 2000 and 2018. Sample sizes ranged from 8 to 149 animals. The waste majority of these studies were performed in rodents (97%). Mortality rates were reported only in nine studies, but no statistically significant risks were identified in these studies between the animals receiving MSCs and the control groups [107]. In terms of efficacy, MSC-treated animals had lower neurological severity scores, and showed superior performances on motor tests compared to the control groups. It appeared that umbilical cord-derived MSC yielded better results than bone marrow-derived or adipose tissue-derived MSCs, and allogeneic or xenogeneic MSCs were superior to autologous MSCs. The optimal time window for MSC administration was between 3 to 30 days post-stroke, with animals receiving transplants > 30 days after stroke showing more modest results in motor and functional recovery [107]. A short overview of the characteristics of the studies analyzed by Lalu and colleagues is provided in Table 2.

#### 3.3.3. Stem Cell Therapies in Clinical Trials for Ischemic Stroke

The basic science and animal models have laid the groundwork for advancing cell-based therapy for ischemic stroke to the clinic. However, ethical issues related to the harvesting of embryonic and fetal neural stem cells, as well as the moratorium on the use of federal funds for research using embryonic stem cells has led to the situation of very few trials using NSCs. A search of cell-based trials for cerebral ischemia among the trials listed in the clinical trials’ database [108] without considering the terminated trials or those whose status is listed as “unknown” yielded only three clinical trials using NSCs, while the vast majority evaluated the safety and efficacy of MSCs (Table 3). A number of the 17 studies have been completed, while 16 are still active or recruiting.

The PISCES trial (NCT01151124), a phase 1 clinical trial during which NSCs were transplanted in increasing doses within 6 months to 5 years after stroke, showed that the intraparenchimatous delivery of the NSC line CTX0E03 is safe, and suggested improvement in neurological function [33], paving the way for a phase 2 trial (PISCES-II, NCT02117635), which transplanted 20 × 10^6^ cells 2 to 3 months post-stroke. Although completed, the results have not been published yet. However, a subsequent clinical phase 2 trial (PISCES-III, NCT03629275) was terminated [108]. Another neural stem cell line, NSI-566 cells, previously used for spinal cord injuries [109] was evaluated in a phase 1 clinical trial (NCT03296618) using intracerebral cell grafts of 1.2–8 × 10^7^ cells with the concomitant administration of immunosuppressants (tacrolimus) for 28 days. It was conducted in China, and its status is currently listed as “unknown” [108], although Zhang and colleagues reported encouraging results on functional scales, and imaging follow-ups revealed new tissue in the post-stroke cavity [34], the nature of which will need further verification.

Despite the positive results reported, the clinical use of NSCs has several drawbacks, such as immunogenicity and the possibility of rejection of allogeneic human NSCs, requiring immunosuppressant therapy, as well as the limited number of donors. Although the rejection of autologous NSCs is uncommon, patients with stroke have a very limited amount of NSCs. A solution to this problem might be the obtaining of iPSC-derived NSCs [110]. In addition, grafting of the stem cells embedded in scaffolds, such as 3D-printed hydrogel scaffolds, increases their survival rate and enables their migration to lesion sites [111].

Mesenchymal stem cells, and especially bone marrow-derived MSCs, are most widely used in clinical trials. Overwhelming evidence supports the safety of the approach, although data on efficacy are sparser or indicate only a transient improvement. The efficacy of autologous bone marrow-derived MSCs administered intravenously 4 weeks after acute ischemic stroke declined by 12 months [112], as did the effect of intravenously delivered autologous bone marrow mononuclear cells (MNCs) within 24–72 h post-stroke after 6 months [113] or the intra-arterial transplant of bone marrow-derived MNCs within 7 days after stroke onset [114]. The great number of trials with MSCs completed or recruiting is listed in Table 3.

It appears that, similar to trials with antioxidants for acute ischemic stroke that yielded encouraging results in animal models but that mostly failed in clinical trials [115], MSCs show the same divergent results. Many reasons may have contributed to this outcome. Most trials enrolled a small number of patients, and some of these were open-labeled. In addition, different donor cells were used in various transplant protocols [19], and the number of transplanted cells was much lower than the number used in animal models. In a preclinical setting the effective dose for intravenous administration was around 4 million cells in rats, which would be equivalent to about 840 × 10^6^ cells in an adult patient, but most trials used a much lower number of cells [19,116]. In addition, gender differences should be also taken into account, as most animal studies used male animal models of stroke. Finally, in human patients, stroke usually occurs in the elderly, and as an epiphenomenon of prior co-morbidities, which should be taken into account when evaluating the efficacy of stem cell therapy in animal models.

#### 3.3.4. Extracellular Vesicles and Exosomes for Ischemic Stroke

Exosomes as natural delivery vehicles are an emerging new avenue in medicine [117]. Being isolated from the patient’s own cells, they are unique tools for personalized medicine. Their surface can be modified to achieve improved targeting, a technology that is used especially in the treatment of cancer [118] or diabetes [119], or they can be loaded with drugs and used for delivery across natural barriers, such as the BBB [118]. In addition, enrichment in certain miRNAs can increase their efficiency in promoting the endogenous reparative processes.

For stroke, there are currently only two trials using extracellular vesicles listed in the database of clinicaltrials.gov [108]: a phase 1/2 trial (NCT05008588) using a conditioned medium with intranasal delivery in addition to umbilical cord-derived MSCs transplanted into the brain parenchyma, and another phase 1/2 trial (NCT03384433) that evaluates the effect of intracerebral delivery of allogeneic MSC-derived exosomes enriched in miR-124 within one month after stroke onset. Both studies are still recruiting patients.

## 4. Future Perspectives

For the efficient bench-to-bedside translation of basic science findings, a well-defined set of phenotypic markers and insight into the mode of action of stem cells, including cell replacement, the secretion of growth factors, and other pathways for promoting endogenous repair processes in the brain, is required. A homogenous population of stem cells or guidelines for the generation of identical populations (Good Manufacturing Practices—GMPs) should be available. Safety procedures regarding the use of fresh or cultured cells, cryopreserved calls, or cellular components (mitochondria, extracellular vesicles, exosomes, or miRNAs) are necessary as well [120,121].

A set of guidelines recommended by the National Institute of Health (NIH) and Food and Drug Administration, together with clinicians, basic scientists, and industry partners under the consortium of Stem Cell Therapeutics as an Emerging Paradigm for Stroke (STEPS), together with NIH and National Institute for Neurological Disorders and Stroke (NINDS) initiative soliciting projects that are aimed at evaluating the potential of neuroprotective drugs in improving the outcomes of approved stroke treatments (the Stroke Preclinical Assessment Network program—SPAN) will hopefully enhance the successful translation of stroke treatments into clinic [19].

Finally, combining stem cell therapy with recanalization procedures or other neuroprotective drugs or biomaterials [122] will hopefully increase the success rates of stem cell therapies and improve recovery in ischemic stroke.

## Figures and Tables

**Table 1:** Overview of the characteristics reported in the studies included by Huang et al. in their meta-analysis of pre-clinical trials with neural stem progenitor cells (NSPCs) for stroke.

Characteristic	Possible Variants	Number of Studies
Stroke model	transient	46
	permanent	16
Animals used in the trial	rats	40
	mice	19
	Mongolian gerbil	2
	pigs	1
Gender	male	54
	female	1
	not stated	7
Source of NSPCs	human	28
	rat	14
	mouse	20
Use of immunosuppressors	yes	21
	no	24
	not stated	14

**Table 2:** Overview of the characteristics reported in the studies included by Lalu et al. in their meta-analysis of pre-clinical trials with mesenchymal stem cells (MSCs) for ischemic stroke.

Characteristics	Possible Variants	Number of Studies
Stroke model	permanent	34
	transient	34
	two arms	4
Animal species	rat	61
	mouse	9
	dog (Beagle)	1
	monkey (Macaca fascicularis)	1
Type of MSCs delivered	bone marrow-derived MSCs	62
	adipose tissue-derived MSCs	2
	umbilical cord- or placenta-derived MSCs	8
Delivery route	intracerebral	37
	intravenous	26
	intra-arterial	4
	intranasal	1
	mixed delivery routes	4

**Table 3:** Clinical trials using stem cell therapies for acute ischemic stroke [108].

Trial Identifier	Phase	Status	Stem Cell Types Used	Protocol
Neural stem cells				
NCT01151124 (PISCES)	1	active, not recruiting	NSCs, CTX0E03 line	Intracerebral delivery of increasing doses 6 months to 5 years post-stroke
NCT02117635 (PISCES-II)	2	completed	Allogeneic NSCs, CTX-derived precursor cells	Intracerebral transplantation of 20 × 10^6^ cells 2–3 months post-stroke
NCT04631406	1	recruiting	Human ESC-derived NR1 cells	Intracerebral graft of increasing doses of cells 6 to 60 months post-stroke
Mesenchymal stem cells				
NCT03080571	1	completed	Autologous BM-derived MSCs	Intra-arterial delivery of an unspecified number of MSCs within 15 days post-stroke
NCT00859014	1	completed	Autologous mononuclear BM-derived stem cells	Intravenous delivery of 10 × 10^6^ cells within 24–72 h post-stroke
NCT004097652	1	completed	Allogeneic UC-derived MSCs	Intravenous delivery of 3 different doses of cells within 48–168 h post-stroke
NCT00473057	1	completed	Autologous BM-MSCs	500 × 10^6^ cells delivered IA in up to 10 patients and IV in up to 5 patients within 3–90 days post-stroke
NCT04434768	1	recruiting	Allogeneic UC-MSCs (UMSC01)	Two arms: intravenous or intravenous *and* intra-arterial delivery following thrombolysis within 36 h after stroke onset
NCT02397018	1	completed	Allogeneic cord blood infusion	0.5–5 × 10^7^ cells/kg within 3–10 days post-stroke
NCT02433509	1	recruiting	Allogeneic human UC-derived monocytes	200–500 × 10^6^ cells delivered as IV infusion within 10 days from stroke onset
NCT01297413	1/2	completed	Allogeneic BM-MSCs	0.5–1.5 × 10^6^ cells/kg delivered IV within 6 months
NCT05292625	1/2	recruiting	Allogeneic UC-MSCs	1.5 × 10^6^ cells/kg delivered IV or intrathecal in stroke patients within 24 months post-stroke, repeated after 3 months
NCT01287936	1/2	completed	Allogeneic modified stem cells (SB623 cell line)	Three arms with doses ranging between 2.5–5 × 10^6^ cells with stereotactic intracerebral delivery 6 to 60 months post-stroke
NCT02605707	1/2	completed	Autologous endothelial progenitor cells	IV, 6 to 60 months post-stroke, number of cells not stated
NCT00535197	1/2	completed	Autologous CD34+ BM-MSCs	Intra-arterial delivery into the ipsilateral MCA within 7 days post-stroke; non-specified number of cells
NCT04608838 (J-REPAIR)	1/2	completed	Allogeneic dental pulp stem cells	1 or 3 × 10^8^ cells delivered IV within 48 h from stroke onset
NCT01468064 (AMETIS)	1/2	completed	Autologous BM and endothelial progenitor cells (EPCs)	Either 2.5 × 10^6^ BM-MSCs or 2.5 × 10^6^ EPCs delivered IV within 4 weeks after stroke onset
NCT04590118 (ASSiST)	1/2	recruiting	Allogeneic human MSCs	0.5 × 10^6^, 1 × 10^6^, or 2 × 10^6^ MSCs delivered IV more than 6 months after stroke onset
NCT03915431	1/2	recruiting	Allogeneic BM-MSCs (NCS-01)	Various number of cells delivered IV within 24 h after stroke onset
NCT04093336	1/2	recruiting	Allogeneic human UC-MSCs	2 × 10^6^ cells/kg transplanted IV within 24 h post-stroke onset
NCT05008588	1/2	recruiting	UC-MSCs + conditioned medium	Intranasal delivery of conditioned medium for 3 days followed by intraparenchymal transplant of 20 × 10^6^ MSCs or just intraparenchymal transplant in ischemic stroke patients (time window not specified)
NCT04811651	2	recruiting	UC-MSCs	IV delivery of 100 × 10^6^ cells in 5 groups: between 6 and 24 h from stroke onset; 1–3 days post-stroke; 4–7 days post-stroke; 1–4 weeks post-stroke; 1–6 months post-stroke
NCT01678534 (AMASCIS-01	2	completed	Allogeneic adipose tissue-derived MSCs	10^6^/kg delivered IV within 2 weeks from stroke onset
NCT02178657	2	active, not recruiting	Autologous BM-MSCs	2 × 10^6^ and 5 × 10^6^ cells/kg delivered IA within 7 days from stroke onset
NCT04280003	2	recruiting	Allogeneic adipose tissue- derived MSCs	10^6^ cells/kg delivered IV within 4 days from stroke onset
NCT02448641 (ACTISSIMA)	2	completed	Modified stem cells (SB623 cell line)	Intraparenchimatous implant of 2.5 × 10^6^ and 5 × 10^6^ cells, 6 to 90 months post-stroke
NCT01501773	2	completed	Autologous BM-MSCs	30–500 × 10^6^ mononuclear cells delivered IV within 7–30 days post-stroke
NCT02425670	2	completed	Autologous BM-MSCs	30–500 × 10^6^ cells injected IV within 7–30 days from stroke onset
NCT03004976(CoBIS2)	2	completed	Allogeneic umbilical cord blood	0.5–5 × 10^7^ cells/kg delivered IV within 3–10 days from stroke onset
NCT00875654 (ISIS)	2	completed	Autologous MSCs	IV delivery within 6 weeks from stroke onset; number of cells not stated, 2 different doses will be used
NCT01436487	2	completed	MULTISTEM investigational adult stem cells	Three different doses of cells delivered IV within 1–2 days from stroke onset
NCT02961504 (TREASURE)	2/3	active, not recruiting	Regenerative cell elements (HLCM051)	1.2 × 10^9^ cells delivered IV 18–36 h after stroke onset
NCT03545607 (MASTERS-2)	3	recruiting	Allogeneic adult stem cells (MULTISTEM)	1.2 × 10^9^ cells infused IV within 18–36 h after stroke onset

NSCs—neural stem cells; MSCs—mesenchymal stem cells; UC—umbilical cord; BM—bone marrow; IV—intravenous; IA—intra-arterial.

## Data Availability

Not applicable.
